# Image-Guided Robotic Radiosurgery for the Treatment of Lung Metastases of Renal Cell Carcinoma—A Retrospective, Single Center Analysis

**DOI:** 10.3390/cancers14020356

**Published:** 2022-01-12

**Authors:** Severin Rodler, Melanie Götz, Jan-Niclas Mumm, Alexander Buchner, Annabel Graser, Jozefina Casuscelli, Christian Stief, Christoph Fürweger, Alexander Muacevic, Michael Staehler

**Affiliations:** 1 Department of Urology, University Hospital of Munich, 81377 Munich, Germany; Melanie.goetz@med.uni-muenchen.de (M.G.); janniclasmumm@med.uni-muenchen.de (J.-N.M.); alexander.buchner@med.uni-muenchen.de (A.B.); annabel.graser@med.uni-muenchen.de (A.G.); Jozefina.casuscelli@med.uni-muenchen.de (J.C.); Christian.stief@med.uni-muenchen.de (C.S.); Michael.Staehler@med.uni-muenchen.de (M.S.); 2 European CyberKnife^®^ Center, 81377 Munich, Germany; christoph.fuerweger@cyber-knife.net (C.F.); alexander.muacevic@cyber-knife.net (A.M.)

**Keywords:** renal cell carcinoma, robotic radiosurgery, metastatic disease, lung metastases

## Abstract

**Simple Summary:**

Patients with metastatic renal cell carcinoma are difficult to treat despite many new systemic therapy options. Patients often present with pulmonary metastases. Local treatment of those metastases is traditionally performed surgically. In this study, robotic radiosurgery is tested in 50 patients with pulmonary metastases and is demonstrated to be a safe and highly effective treatment option in this patient group. Future research might focus on the combination of robotic radiosurgery with systemic treatment.

**Abstract:**

Pulmonary metastases are the most frequent site of metastases in renal cell carcinoma (RCC). Metastases directed treatment remains an important treatment option despite advances in systemic therapies. However, the safety and efficacy of robotic radiosurgery (RRS) for the treatment of lung metastases of RCC remains unclear. Patients with metastatic RCC and lung metastases treated by RRS were retrospectively analyzed for overall survival (OS), progression-free survival (PFS), local recurrence free survival (LRFS) and adverse events. The Kaplan–Meier method was used for survival analysis and the common terminology criteria for adverse events (CTCAE; Version 5.0) classification for assessment of adverse events. A total of 50 patients were included in this study. Median age was 64 (range 45–92) years at the time of RRS. Prior to RRS, 20 patients (40.0%) had received either tyrosine kinase inhibitors or immunotherapy and 27 patients (54.0%) were treatment naïve. In our patient cohort, the median PFS was 13 months (range: 2–93). LRFS was 96.7% after two years with only one patient revealing progressive disease of the treated metastases 13 months after RRS. Median OS was 35 months (range 2–94). Adverse events were documented in six patients (12%) and were limited to grade 2. Fatigue (*n* = 4) and pneumonitis (*n* = 2) were observed within 3 months after RRS. In conclusion, RRS is safe and effective for patients with metastatic RCC and pulmonary metastases. Radiation induced pneumonitis is specific in the treatment of pulmonary lesions, but not clinically relevant and survival rates seem favorable in this highly selected patient cohort. Future directions are the implementation of RRS in multimodal treatment approaches for oligometastatic or oligoprogressive disease.

## 1. Introduction

Renal cell carcinoma (RCC) is a heterogenous disease accounting for 4% of all newly diagnosed cancers [1]. For localized disease, surgical intervention is recommended including kidney sparing approaches [2]. For metastatic disease, the advances of systemic therapies have had a major impact on current guidelines. Tyrosine kinase inhibitors (TKI) as well as immunotherapy have shown clinical efficacy and are established as the standard of care either as combination therapies, especially in a first-line setting [3,4] or as single agents in subsequent therapeutic lines [5,6]. Despite major advances in systemic therapeutic options, local therapies remain an important option that is applied in selected patients [7].

The treatment of lung metastases is of high interest in patients with RCC, as the lungs are the most frequent metastatic site at diagnosis of metastatic disease of RCC [8]. Conventional radiological techniques are limited in their efficacy as high doses are required in renal cell carcinoma due to a relative radio resistance [9]. Pulmonary tissue is sensitive to radiation and radiation-induced lung injuries as pneumonitis or fibrosis limit the applied doses and thereby impact local tumor control rates [10]. Several studies have investigated the safety and efficacy of stereotactic body radiotherapy (SBRT) modalities including robotic radiosurgery (RRS) [11,12,13]. However, study cohorts were highly heterogenous and patients presented with metastases originating from various primary tumors. RCC and other tumors reveal different biological behavior [14]. In addition, real world evidence from RCC cohorts is required to guide therapeutic decision making in the light of current therapeutic options.

We aimed to investigate the approach of RRS in metastatic RCC with lung metastases. Therefore, overall survival (OS), progression-free survival (PFS) as well as adverse events were analyzed in a cohort of patients with RCC and lung metastases.

## 2. Results

Between 2009 and 2021, 50 patients with metastatic RCC underwent RRS treatment for lung metastases and received follow-up at our academic center. Median age of all patients was 64 (range 45–92) years at the time of RRS. A total of 49 patients (98.0%) presented with clear cell RCC and only one patient (2.0%) with papillary type 1 RCC. A total of 48 patients (96%) had received partial or radical nephrectomy previously, and two patients revealed metastatic disease at first diagnosis. At the time of RRS, 20 patients (40.0%) received either tyrosine kinase inhibitors or immunotherapy. A total of 27 patients (54.0%) were treatment naïve. Median follow-up time of all patients was 22.5 (range 1–117) months (Table 1).

RRS was performed in a hypofractionated high-dose regimen. A median of one pulmonary metastasis (range 1–2) was treated. The median number of fractions was one (range 1–5) with nine patients receiving RRS split into five doses and one patient receiving RRS split into three doses. The median target volume was 23.6 (range 4.9–225.4) cm^3^ (Table 2).

**Figure 1:**
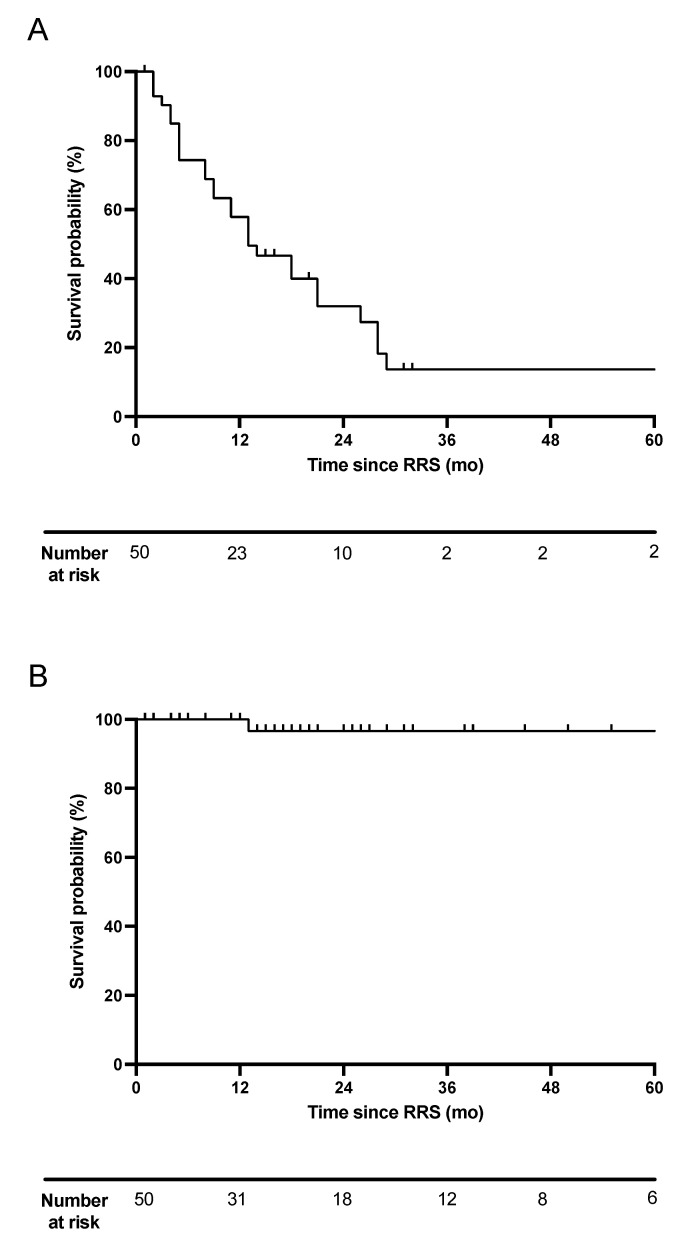
Progression-free survival and local progression-free survival after RRS. (**A**): Progression-free survival was calculated by Kaplan–Meier method. (**B**) Local progression-free survival was defined as recurrence within the area of the previous RRS. RRS: robotic radiosurgery, mo: months.

Following RRS treatment, median progression-free survival (PFS) was 13 months (range: 2–93) (Figure 1A). Progression after RRS was observed in 29 patients (58%), with lungs (*n* = 11), lymph nodes (*n* = 6) and bone (*n* = 6) as the most frequent sites of progression. One patient progressed in pulmonary and lymph node metastases after RRS. Further sites of progression were brain (*n* = 2), esophagus (*n* = 2), pancreas (*n* = 1), spinal cord (*n* = 1) and adrenal glands (*n* = 1) (Table 3). Local progression-free survival of the RRS treated metastases was 96.7% after two years. Only one patient (2%) progressed locally 13 months after RRS (Figure 1B).

**Figure 2:**
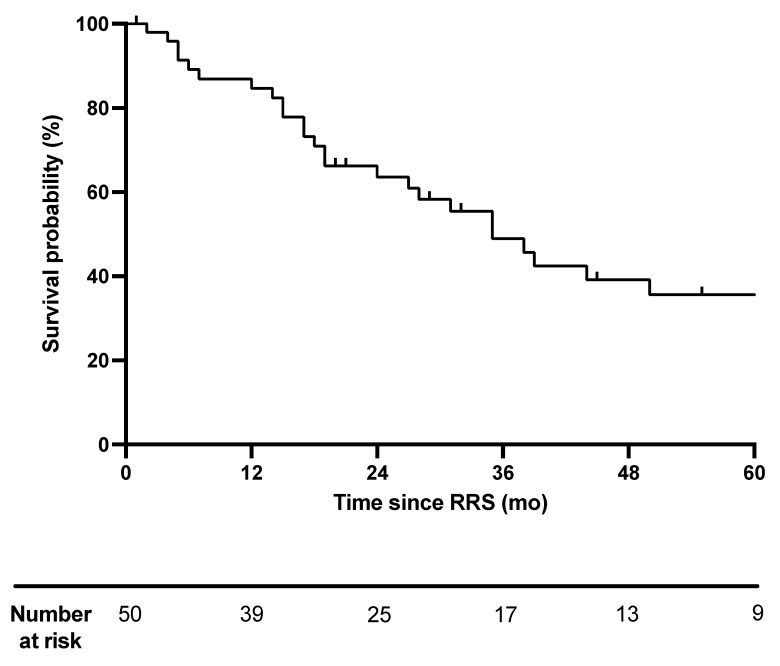
Overall survival after robotic radiosurgery. Overall survival (OS) was calculated with Kaplan–Meier method and is depicted in months (mo). OS is calculated from the time of robotic radiosurgery (RRS) until death (event) or loss to follow-up (censored). RRS: robotic radiosurgery, mo: months.

Median OS was 35 months (range 2–94). After two years, 25 patients were still at risk with a 63.6 survival probability (Figure 2).

Adverse events were observed in six patients (12%). Acute adverse events were limited to grade 2. The most frequent adverse events were fatigue (*n* = 4) and pneumonitis (*n* = 2). Fatigue was limiting instrumental activities of daily living in one patient (grade 2), whereas pneumonitis was only detected in CT controls and was not clinically relevant. There was no additional RRS related late toxicity observed in the study cohort (Table 4).

## 3. Discussion

Our study reveals a clinically meaningful efficacy and safety of RRS in patients with pulmonary metastases of RCC. Local tumor control was excellent with 96.4% after 2 years. In addition, local treatment of pulmonary metastases was safe and was associated with low toxicity with grade 2 CTCAE as the most severe adverse event. Pneumonitis occurred as the tissue specific adverse event of RRS treatment of pulmonary metastases. PFS and OS seems promising in this highly selected patient cohort.

Thus far, it remains unclear how patient centered approaches ranging from routine molecular testing to tailored systemic therapy regimens integrating multimodal approaches affect outcomes of patients [15,16]. In RCC, local treatment has been part of the treatment armamentarium for many years as part of multimodal approaches. Metastasectomy has traditionally played an important role. Through advances in radiation technology, RRS was introduced as a treatment alternative. The specific efficacy of RRS in pulmonary tissue and adverse events associated with this high dose hypofractionated radiotherapy has been discussed in oligometastatic patients but gains interest in several other indications as oligoprogressive disease under concomitant systemic therapy [17].

RRS has been proven to be effective in RCC. Correa et al. have shown in a systematic review that the local tumor control by stereotactic body radiotherapy (SBRT) is 97.2% with a rate of 1.5% of grade 3 or 4 adverse events in primary renal tumors [18]. However, treatment of the primary tumor has mainly been used as a treatment alternative in frail patients with high morbidity or risk of renal failure. In oligometastatic RCC, a 1-year local tumor control rate of 89.1% was observed across 11 studies with various metastatic sites [19]. As results vary throughout the studies and highly heterogenous cohorts have been analyzed, several studies focus on the efficacy of RRS in specified tissue.

For other sites as lymph nodes and visceral metastases, we demonstrated high local recurrence free survival rates in a contemporary cohort [20]. Stereotactic radiotherapy in pulmonary metastases has been shown to have 3 year control rates of 91.9%. Interestingly, higher doses showed a trend towards better outcomes in this study [21]. Recent clinical trial data revealed that single-fraction SBRT is more effective than multi-fraction SBRT in pulmonary oligometastases [22]. This evidence supports the rationale for RRS treatment in the setting of renal cell carcinoma with pulmonary metastases. We observe a 2-year progression-free survival in our study of 96.7%.

Our data prove that a local efficacy of RRS similar to treatment at other metastatic sites can be reached in pulmonary tissue. As local control is high, the safety of RRS for the treatment of pulmonary metastases has to be considered.

Adverse events depend on the tissue that is treated by radiotherapy [23]. The overall rates of grade 3–5 adverse events in SBRT for treatment of RCC with metastatic disease is 0.7% for extracranial metastases [19]. In contrast, alternative minimal invasive local treatment options such as radiofrequency ablation show 3% major complication rates [24]. Thus far, the standard for local control of pulmonary metastases was metastasectomy [2]. Complication rates in primary metastasectomy of lung metastases of RCC are 4%, with pneumonia as one of the most frequent adverse events [25]. In our study, we observed radiologically detected pneumonitis that was specific to RRS treatment. However, it was clinically asymptomatic and therefore required no further treatment.

Local treatment of pulmonary metastases as part of a multimodal therapy has gained interest in recent years. Metastasectomy still plays a role in this setting and is used in oligometastatic disease [2]. In this setting, surgery is used to achieve a complete resection to avoid or postpone systemic treatment. Parallel to the advancements in immunotherapy based systemic therapies, treatment of oligoprogressive disease has gained the attention of clinicians [17]. Here, surgery can cause immunosuppression and, therefore, might have worse outcomes [26]. RRS, on the other hand, might stimulate an innate and adaptive immune response through release of damage-associated molecular patterns and neoantigens, and therefore harbor additive effects [9]. RRS is used equivalently to metastasectomy to enhance response rates and to prolong therapy lines. This hypothesis has been tested in several trials. In the phase I/II Rapport trial, the median PFS was 15.6 months in patients with oligometastatic RCC treated with pembrolizumab and SBRT [27]. In the phase II NIVES study, the combination of immunotherapy and SBRT in second or later line treatment in mRCC has not revealed a survival benefit. However, the study protocol only applied 10 Gy in 3 fractions [28]. Another limiting factor is the radiological assessment of RRS treated lesions. RECIST criteria mainly focus on the size of lesions [29]. However, RRS induced apoptosis in the treated tissue and lesions can be detectable in terms of size despite not showing perfusion [30,31].

Despite all limitations of the described trials, future research and application of RRS and SBRT in mRCC might focus on oligoprogressive disease during systemic therapy [17]. In this setting, RRS is not used at the initiation of a systemic treatment line as described, but at the end in order to prolong the time on this therapy line and extend PFS, to reduce toxicity of further therapy lines and ultimately to prolong OS. A prospective study assessing SBRT in oligoprogressive disease under TKI therapy revealed a median PFS of 9.3 months and a change to a subsequent therapy at a median time of 12.6 months [32]. The survival data of our study with a median PFS after RRS of 13 months and median OS of 35 months is comparable and further supports the rationale to explore prospectively the use of RRS in oligometastatic and oligoprogressive patients with metastatic RCC.

As we present a single-center, retrospective study on RRS treatment of pulmonary metastases, several limitations have to be considered. Selection bias is a major problem in all retrospective RRS studies, as they tend to enclose patients with good prognosis features. Therefore, PFS and OS data has to be interpreted in light of this problem. Further, the study period is enclosing patients between 2009 and 2021 and, therefore, patients with different subsequent therapeutic options in later therapy lines. However, we present one of the largest studies focusing on RRS in pulmonary metastases. In addition, we used high ablative doses with a detailed prescription plan for every patient included in this study. Thereby, we ensure a high methodological accuracy, that might be lacking in older studies with varying doses.

## 4. Materials and Methods

We retrospectively analyzed patients undergoing RRS at the European Cyberknife Center. All patients were followed up at our academic center with a specialized RCC outpatient clinic. Inclusion criteria for this study was histologically confirmed RCC, radiologically detected metastases and RRS treatment of pulmonary metastases. Five or fewer metastatic lesions were defined as oligometastatic disease. Patients were allowed to have received prior systemic therapies for RCC.

The Cyberknife robotic radiosurgery system (Accuray Inc., Sunnyvale, CA, USA) was used for all RRS treatments. RRS treatments were performed as outpatient procedures. All treatment plans were generated using a Monte Carlo dose calculation algorithm for tissue heterogeneity correction. The treatment dose was prescribed to the margin of the PTV, which was defined as the visible solid tumor with an isotropic expansion of 4 to 6 mm [33]. As previously described, a 6MV linear accelerator is moved into treatment positions by a high-precision robot arm. A typical therapy session consists of 100 to 120 radiation beams that are delivered from 270 degrees around the patient. Organ movement is detected in real-time and therefore radiation beams are adjusted accordingly [20]. Multiple fractions were delivered on consecutive days. When patients were on a concomitant systemic therapy during RRS treatment, the systemic therapy was continued until disease progression.

Follow-up was performed at our academic center as part of an outpatient clinic specialized in treatment of localized and metastatic RCC. Here, patients were counseled regarding tumor response and adverse event management. Tumor response was evaluated by board certified radiologists according to the response evaluation criteria in solid tumors (RECIST) version 1.1 [29]. Patients undergoing systemic therapy received a contrast enhanced CT staging of the thorax and abdomen every three months and a brain MRI/CT once per year. Treatment response was classified according to the RECIST-criteria. RRS related adverse events were analyzed and classified according to the National Cancer Institute Common Terminology Criteria for Adverse Events (CTCAE version 5.0). Acute adverse events occurring within the first 90 days after RRS were included.

For calculation of overall survival (OS), progression-free survival (PFS) and local recurrence free survival (LRFS) we used the Kaplan–Meier method. OS was calculated from the date of RRS until death or loss to follow-up. PFS was measured from the date of RRS until the first CT showing progressive disease irrespective of the site of progression according to RECIST 1.1 or loss to follow-up. LRFS was calculated from RRS until local progression of the radiated lesion or loss to follow-up. For statistical analysis we used Graphpad Prism Software (Version 9.0, San Diego, CA, USA).

Prior to initiation of the study we received approval for the study design by the local ethics authorities (Ethikkomission der Ludwig-Maximilian-Universität München, reference number: 20-1092).

## 5. Conclusions

RRS is a safe and effective for the treatment of pulmonary metastases of patients with metastatic RCC. Local tumor control is excellent, and tissue specific adverse events of radiation-induced pneumonitis are limited to grade 1 and, therefore, not clinically relevant. As outcomes of this highly selected patient cohort seem promising, further studies should focus on the implementation of RRS in multimodal treatment approaches for oligometastatic and oligoprogressive disease.

## Figures and Tables

**Table 1:** Patient characteristics.

Parameter	(*n* = 50)	
	%	*n*
Age at diagnosis		
Median	55	
Range	40–88	
Age at RRS treatment		
Median	64	
Range	45–92	
Gender		
Male	74.0	37
Female	26.0	13
IMDC *		
Favorable	69.4	34
Intermediate	26.5	13
Poor	4.1	2
Histology		
Clear cell	98.0	49
Papillary type 1	2.0	1
Prior therapies		
Surgery	96.0	48
TKI	40.0	20
IO	18.0	9
RRS	24.0	12
Oligometastatic disease at the time of RRS		
Yes	64.0	32
No	36.0	18
Concomitant therapy during RRS		
No systemic therapy	58.0	29
TKI therapy	28.0	14
IO therapy	10.0	5
TKI-VEGF/IO therapy	4.0	2
Number of systemic therapy lines prior or at RRS		
1	69.6	16
2	17.4	4
≥3	13.0	3
Sites of metastases prior to or at RRS		
Pulmonary	100.0	50
Lymph node	42.0	21
Brain	14.0	7
Bone	10.0	5
Pancreas	10.0	5
Liver	8.0	4
Adrenal gland	4.0	2
Esophagus	2.0	1
Soft tissue	2.0	1
Spleen	2.0	1

* The patient with non-clear cell histology has not been included in IMDC risk analysis. Abbr.: RRS: robotic radiosurgery, IMDC: international metastatic renal cell carcinoma database, TKI: tyrosine kinase inhibitor, IO: immune oncology.

**Table 2:** Robotic radiosurgery treatment parameters.

Parameter	Patients with Lung Metastases	
	(*n* = 50)	
	Median	Range
Metastases	1	1–2
Fractions	1	1–5
Prescription dose (Gy)	26	22–45
Prescription isodose (Gy)	70	55–75
Target volume (cm^3^)	23.6	4.9–225.4

**Table 3:** Site of progression after RRS.

Site of Progression after RRS	%	*n*
Lung *	1	11
Lymph nodes *	26	6
Bone	70	6
Brain		2
Esophagus		2
Pancreas		1
Spinal cord		1
Adrenal gland		1

* 1 patient progressed in mediastinal lymph nodes and the lung.

**Table 4:** Acute adverse events.

CTCAE Term	Grade 1	Grade 2
Fatigue	3	1
Pneumonitis	2	0

CTCAE: Common Terminology Criteria for Adverse Events.

## Data Availability

The data presented in this study are available on request from the corresponding author.
